# Ultrastructural Evidence of Mitochondrial Dysfunction in Osteomyelitis Patients

**DOI:** 10.3390/ijms24065709

**Published:** 2023-03-16

**Authors:** Daniel H. Mendelsohn, Tanja Niedermair, Nike Walter, Volker Alt, Markus Rupp, Christoph Brochhausen

**Affiliations:** 1 Institute of Pathology, University Regensburg, 93053 Regensburg, Germany; 2 Central Biobank Regensburg, University Regensburg, University Hospital Regensburg, 93053 Regensburg, Germany; 3 Department of Trauma Surgery, University Medical Centre Regensburg, 93053 Regensburg, Germany; 4 Institute of Pathology, University Medical Centre Mannheim, 68167 Mannheim, Germany

**Keywords:** osteomyelitis, mitochondria, mitochondrial ultrastructure, mitochondrial dysfunction, mitochondrial dynamics

## Abstract

Osteomyelitis is a difficult-to-treat disease with high chronification rates. First studies suggest increases in mitochondrial fission and mitochondrial dysfunction as possible contributors to the accumulation of intracellular reactive oxygen species and thereby to the cell death of infected bone cells. The aim of the present study is to analyze the ultrastructural impact of bacterial infection on osteocytic and osteoblastic mitochondria. Human infected bone tissue samples were visualized via light microscopy and transmission electron microscopy. Osteoblasts, osteocytes and their mitochondria were analyzed histomorphometrically and compared with the control group of noninfectious human bone tissue samples. The results depicted swollen hydropic mitochondria including depleted cristae and a decrease in matrix density in the infected samples. Furthermore, perinuclear clustering of mitochondria could also be observed regularly. Additionally, increases in relative mitochondrial area and number were found as a correlate for increased mitochondrial fission. In conclusion, mitochondrial morphology is altered during osteomyelitis in a comparable way to mitochondria from hypoxic tissues. This gives new perspectives on the treatment strategies since the manipulation of mitochondrial dynamics may improve bone cell survival as a potential new target for the therapy of osteomyelitis.

## 1. Introduction

First described by Hippocrates, osteomyelitis is the infection of bone and bone marrow accompanied by bone destruction and new bone formation [1]. Despite its ancient history and generations of surgeons being faced with this recalcitrant disease, bacterial osteomyelitis remains a challenging disease to treat. Chronification rates are very high and sometimes, after multiple surgical debridements, the last reasonable treatment option for the patient is the amputation of the afflicted limb [2].

Despite efforts to improve diagnostics and treatment methods, especially within the last century, the pathogenesis of chronic osteomyelitis is still incompletely understood. Costerton described and coined the word biofilm, which can be regarded as a milestone in the understanding of the pathophysiology of the disease [3]. The biofilm hypothesis describes the formation of biofilms on dead or foreign matter. These in most cases polymicrobial hydrophobic “fortresses” display immense resistance towards antibiotics which makes chronic osteomyelitis a disease that must be treated surgically. Biofilms have to be removed to achieve infection eradication [2].

Another pathophysiological theory is the internalization of bacteria into osteoblasts and osteocytes [4]. Thus, bacteria can hide from and evade the immune system and replicate intracellularly. For *Staphylococcus aureus* (*S. aureus*)—the most common etiologic agent in chronic bacterial osteomyelitis—the formation of so-called small-colony variants (SCVs) has been reported [5]. SCVs are less susceptible to antibiotics than their larger phenotypes. The clinical evidence around internalized *S. aureus* in osteomyelitis, however, is restricted to one sole case report [6]. 

Due to the obvious direct interconnection between bone cells and infection-causing bacteria, mitochondrial dynamics and function in bone cells following infection have gained increasing attention [7,8]. Mitochondria are responsible not only for energy production, but also for several vital processes such as calcium homeostasis, cellular immunity and even cell death or apoptosis in eukaryotic cells [9]. Hence, it is no surprise that these organelles are involved in health and disease, which became increasingly evident in the past decades [10,11]. Mitochondrial dysfunction is connected to a variety of pathologic conditions including neurodegenerative diseases such as Parkinson’s or Alzheimer’s disease [12,13], as well as diabetes and chronic inflammation [14,15]. Furthermore, it plays an important role in infectious diseases because pathogens can exploit mitochondria to reduce immunity and even initiate apoptosis. This has also been shown for the *S. aureus* toxin Panton–Valentine leukocidin (PVL) that directly targets mitochondria and induces apoptosis in neutrophil granulocytes [16]. Additionally, microenvironmental tissue hypoxia is typical in infections including osteomyelitis [17]. Meanwhile, mitochondria are the main source of oxidative stress and cell damage during hypoxia [18]. 

Mitochondria are no longer regarded as static singular organelles, but rather as an ever-changing dynamic network [19]. All the dynamic processes that allow mitochondria to adapt to the physiological needs of the cell are summarized by the term mitochondrial dynamics. The main processes are mitochondrial fusion, fission (or division) and autophagic clearance of mitochondria, which is known as mitophagy. While fusion allows a short-term increase in mitochondrial efficiency, fission is required for the transfer of mitochondria to intracellular compartments with increased energy demands and to sort out defective mitochondria before their mitophagic clearance [20,21,22]. In the case of excessive fission or mitochondrial fragmentation, as is the case in chronic stress conditions such as hypoxia, the mitophagic system is oversaturated and defective mitochondria accumulate, producing large amounts of reactive oxygen species (ROS) and subsequently leading to cellular damage and cell death [23,24]. The exact molecular mechanisms involved in mitochondrial dynamics have been thoroughly studied from yeast to numerous mammalian cell lines [19,25,26]. They are a hot topic, especially in energy-intensive tissues such as myocardial or nerve tissue. The first medical agents targeting mitochondrial fission have been shown to reduce reactive oxygen species production and alleviate cellular stress [27]. In orthopaedics and trauma surgery, vastly less is known about mitochondrial function and dynamics. A gene expression analysis of broilers with bacterial chondronecrosis suggested an increase in mitochondrial fission and dysfunction following infection [7]. Due to mitochondrias’ prominent involvement in chronic osteomyelitis, it is vital to further investigate this aspect and deepen our understanding of the disease. 

Therefore, we aim (1) to qualitatively analyze mitochondrial morphology in bone cells from human samples from patients with chronic osteomyelitis via transmission electron microscopy, (2) to establish a method to quantitatively analyze mitochondrial fission and fusion parameters in bone specimens and compare them with healthy bone samples, and (3) to quantify the prevalence of intracellular bacterial pathogens in the examined samples.

## 2. Results

Samples were acquired from 22 patients, of which 14 could be included in the infectious group and four in the control group. The remaining four patients were excluded due to a lack of vital bone cells in the samples. The mean age in the infectious group was 55.79 years (S.D. = 19.28 years). The mean age of the control group was 57.25 years (S.D. = 11.10 years). Both groups are summarized in Table 1. Comorbidity is expressed via the Charlson Comorbidity Index (CCI) [28]. In total, 72 osteoblasts from the infectious group were compared with 16 osteoblasts from the control group, and 93 osteocytes from the infectious group were compared with 34 osteocytes from the control group.

**Figure 1:**
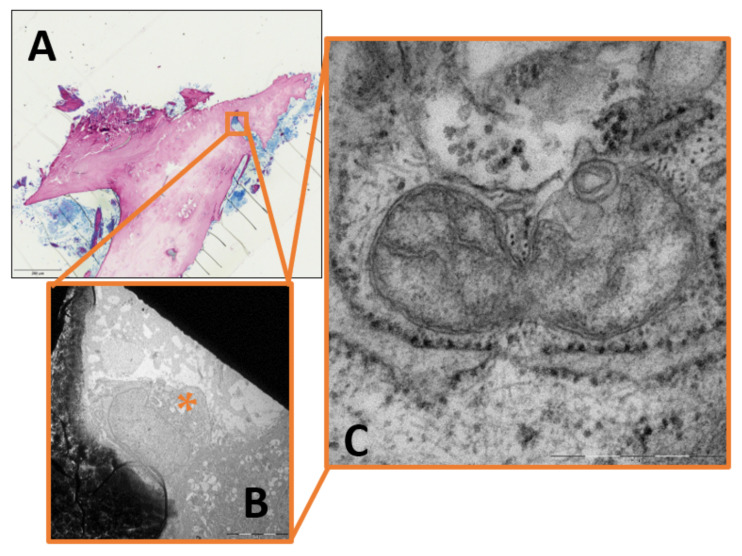
(**A**) Light microscopy image of infectious bone (magnification × 40). (**B**) Magnification of (**A**). Transmission electron microscopy image of osteoblast next to osteoclast. Fission site indicated by star (magnification × 5000). (**C**) Magnification of (**B**). Mitochondrion in midst of fission that is being pinched by endoplasmic reticulum (magnification × 40,000).

**Figure 2:**
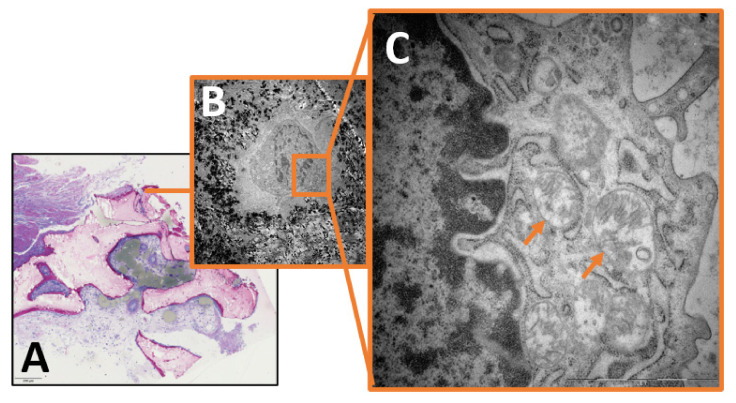
(**A**) Light microscopy image of infectious bone (Magnification × 40). (**B**) Magnification of (**A**). Transmission electron microscopy image of osteocyte from patient with osteomyelitis (Magnification × 5000). (**C**) Magnification of (**B**). Transmission electron microscopy image of perinuclear region of osteocyte. Visible cluster of swollen mitochondria with depleted cristae and reduced matrix density. Mitochondria exemplarily indicated by arrows (magnification × 20,000).

**Figure 3:**
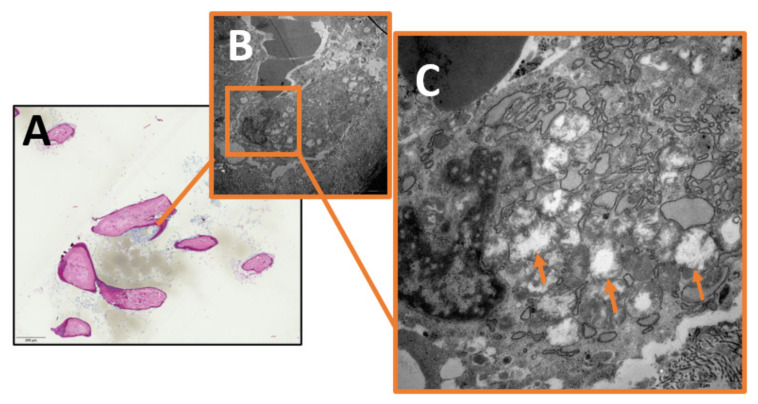
(**A**) Light microscopy image of infectious bone (magnification × 40). (**B**) Magnification of (**A**). Transmission electron microscopy image of osteoblast from patient with osteomyelitis (Magnification × 5000). (**C**) Magnification of (**B**). Transmission electron microscopy image of perinuclear region of osteoblast. Visible cluster of swollen mitochondria with depleted cristae and reduced matrix density. Mitochondria exemplarily indicated by arrows (magnification × 20,000).

**Figure 4:**
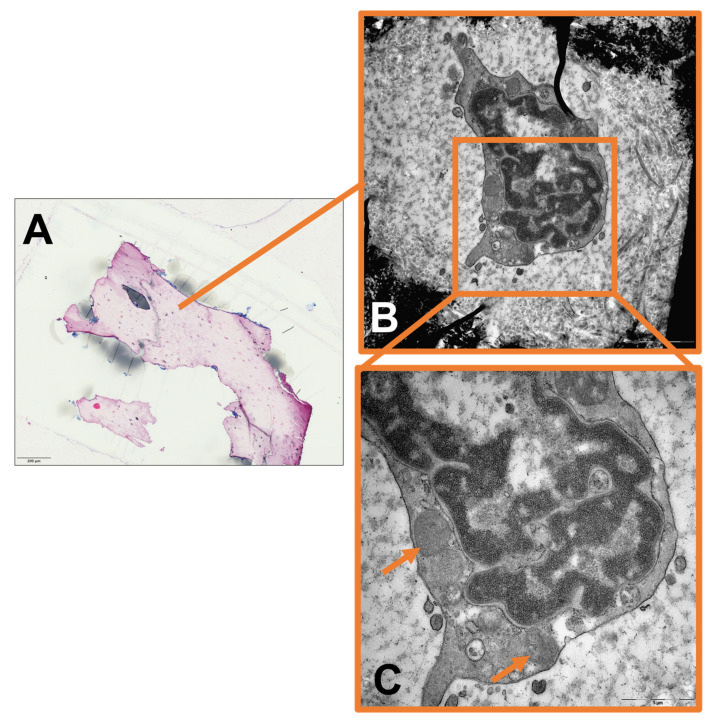
(**A**) Light microscopy image of non-infectious bone for comparison (magnification × 40). (**B**) Magnification of (**A**). Transmission electron microscopy image of non-infectious osteocyte (magnification × 5000). (**C**) Magnification of (**B**). Transmission electron microscopy image of perinuclear region of osteocyte. Visible solitary mitochondria with sustained cristae and matrix density indicated by arrows (magnification × 20,000).

Qualitative image analysis showed that mitochondrial pinching events could be seen in the infectious group as a morphological correlate of ongoing mitochondrial fission, but were absent in the control group (Figure 1). Furthermore, mitochondria from the infectious group tended to appear swollen or hydropic, with depleted cristae and a reduction in matrix density. Large clusters of these hydropic mitochondria could often be found in the perinuclear region (Figure 2, Figure 3 and Figure 4).

**Figure 5:**
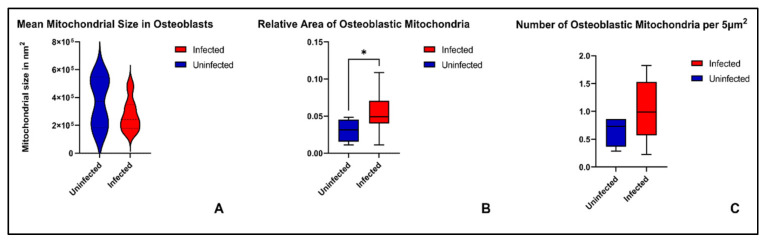
(**A**) Violin plot of mean mitochondrial sizes in osteoblasts, measured in nm^2^ and showing no measurable difference between groups. (**B**) Box plot of relative cellular area occupied by mitochondria in osteoblasts showing significant increase in the infected group. * Indicates significant difference at *p* < 0.05. (**C**) Box plot of number of mitochondria per 5µm^2^ in osteoblasts showing non-significant increase in the infected group.

Quantitative analysis of mitochondria achieved significant results for both cell types. In osteoblasts, the mean area of single mitochondria was 274,410 nm^2^ (S.D. = 114,598 nm^2^) in the infectious group compared with 367,895 nm^2^ (S.D. = 189898 nm^2^) in the non-infectious group. However, the mean percentage of cellular area occupied by mitochondria in the infectious group was 5.56% (SD = 2.60%) compared with 3.06% (S.D. = 1.55%) in the non-infectious group and thus, significantly higher (*p* = 0.0431). The comparison of the mean number of mitochondria per 5 µm^2^ showed similar results. With 1.04 (S.D. = 0.52) in the infectious and 0.65 (S.D. = 0.24) in the non-infectious group, the number of osteoblastic mitochondria was increased following infection. This was seen in a strong but non-significant tendency (*p* = 0.0754) (Figure 5).

**Figure 6:**
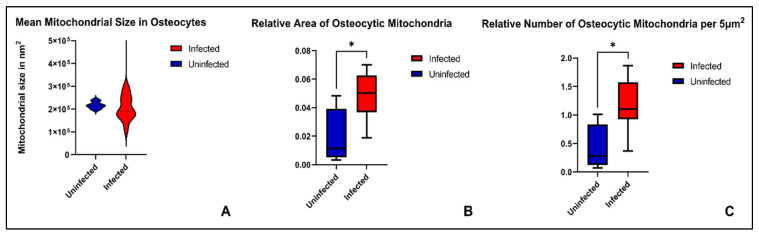
(**A**) Violin plot of mean mitochondrial sizes in osteocytes, measured in nm^2^ and showing no measurable difference between groups. (**B**) Box plot of relative cellular area occupied by mitochondria in osteocytes showing a significant increase in the infected group. (**C**) Box plot of number of mitochondria per 5 µm^2^ in osteocytes showing a significant increase in the infected group. * Indicates significant difference at *p* < 0.05.

The osteocytic mitochondria had a mean area of 288,512 nm^2^ (S.D. = 302,729 nm^2^) in the infectious versus 217,261 nm^2^ (S.D. = 15,607 nm^2^) in the non-infectious group, and thus there was no significant difference. Concerning the mean percentage of cellular area occupied by mitochondria and the mean number of mitochondria per 5 µm^2^ in osteocytes, there were significant increases in the infectious samples. The relative area occupied was 4.93% (S.D. = 1.62%) in the infectious and 1.87% (S.D. = 2.02%) in the non-infectious group (*p* = 0.0468). The mean number of mitochondria per 5 µm^2^ was 1.19 (S.D. = 0.43) in the infectious and 0.41 (S.D. = 0.41) in the non-infectious group (*p* = 0.0214) (Figure 6).

Regarding intracellular bacteria, neither in the histological slides nor in the electron microscopic images could any internalized bacteria be found.

## 3. Discussion

### 3.1. Mitochondrial Dysfunction and Oxidative Stress in Chronic Osteomyelitis

In the present study, we detected hydropic or swollen mitochondria with reduced matrix densities and depleted cristae in association with the concentration of mitochondria in the perinuclear region in the infectious bone samples. These are morphological signs of mitochondrial damage [29], which are in concordance with the findings of Ferver and colleagues, who postulated mitochondrial dysfunction through an upregulation of mitochondrial biogenesis-associated genes in tissues from bacterial chondronecrosis with osteomyelitis-affected broilers [7]. Mitochondrial damage is associated with the depolarization of the mitochondrial membrane potential and a subsequent opening of permeability transition pores and hydropic mitochondrial swelling [30]. This diminishes the electron transport chain activity and induces the production of ROS [31], hence enhancing mitochondria-mediated cell death [32]. Interestingly, ultrastructural analysis of cardiac mitochondria following hypoxia showed similar results [32,33], which further emphasizes the involvement of microenvironmental tissue hypoxia in chronic osteomyelitis [17,34,35]. Chronic cellular stress leads to the accumulation of defective proteins, for example, of respiratory chain complexes [35]. Mitochondria containing defective proteins produce ROS in large amounts, which then leads to further cellular damage [36,37]. ROS themselves activate a series of genes responsible for the cells’ oxidative response [38]. Thus, defective mitochondria are transported to the perinuclear region via microtubules before degradation [24], so their emitted ROS can reach the nucleus without causing excessive collateral damage in the cell. Among others, one pathway that ROS induce is mitochondrial fission itself. Fission allows defective mitochondria to be sorted out. If these cannot be cleared fast enough, they cluster perinuclearly and produce further ROS. This leads to a self-perpetuating cycle of fission and ROS production and ultimately cell death [23].

Several antioxidant treatment strategies to ameliorate mitochondrial function are being developed [39]. However, these mainly focus on neurodegenerative diseases and cardiomyopathy. One promising new treatment method is mitochondrial transplantation [40,41]. It has been demonstrated that mitochondrial injection preceding reperfusion of damaged brain or heart tissue can alleviate the symptoms of ischemia/reperfusion injury [42]. Damaged mitochondria are the main source of oxidative stress-induced cell damage during reperfusion [43,44,45]. Healthy autologous mitochondria can be internalized into cells via endocytosis and fused with the mitochondrial network [46], improving the overall mitochondrial function of the cell [47,48]. This suggests that mitochondrial transplantation could improve mitochondrial function in bone cells as well, especially if it precedes hyperbaric oxygen therapy [49]. To date, no studies have been performed on the possibility of mitochondrial transplantation in bone cells. It is vital to assess the possible value of mitochondrial transplantation in orthopaedic diseases.

### 3.2. Mitochondrial Fragmentation in Chronic Osteomyelitis

Quantitative analysis showed an increase in mitochondrial number and in cellular area occupied by mitochondria in osteoblasts and osteocytes following infection. In concordance with Ferver’s findings [7] and the evidence of mitochondrial pinching events in the infectious group (Figure 1), this indicates a shift of mitochondrial fission and fusion towards fission. Imbalances in mitochondrial dynamics such as mitochondrial fragmentation are associated with mitochondrial damage. The subsequent release of ROS and mitochondrial contents lead to damage-associated molecular patterns (DAMPs) such as mtDNA and cardiolipin being released into the cytosol and outside the cell [50]. Via an activation of nuclear factor-kappaB (NF-κB), inflammation can be induced by ROS [51,52]. DAMPs can trigger inflammation and elicit innate immunity [50]. Together with the connection to cell death and apoptosis, it becomes clear why mitochondrial fission could play a pivotal role in chronic osteomyelitis.

A common aspect of osteomyelitis is impeded bone mineralization and extensive bone tissue necrosis [53]. Interestingly, an increase in mitochondrial fission in osteoblasts is also linked to an increase in the ratio of receptor activator of nuclear factor-kappaB ligand (RANKL) to osteoprotegerin (OPG) [54]. The RANKL/OPG ratio is a determinant of bone mass and skeletal integrity [55]. Thus, increased fission is connected to additional bone destruction, which furthermore suggests that the inhibition of fission might offer promising results in the treatment of osteomyelitis. As aforementioned, microenvironmental tissue hypoxia is being increasingly linked to bacterial infection, which becomes evident through an upregulation of hypoxia-inducible factor 1-alpha (HIF-1a). The inhibition of HIF-1a is thought to be a novel strategy in treating bacterial infections. This has also been demonstrated for *S. aureus*-caused osteomyelitis [17]. A recent study by Zhang and colleagues elucidates the role of upregulated HIF-1 and consequent transforming growth factor-β1 (TGF-β1) in osteomyelitis and its subsequent impaired osteogenesis and bone mineralization. They also suggested that inhibition of HIF-1 reduces inflammation markers and restores bone mineralization [17]. This contradicts the findings of Sasaki and colleagues, who demonstrated that HIF-1 is upregulated in spontaneous wound healing and bone repair in a mouse model resembling osteomyelitis [56]. Since mitochondrial fission agent dynamin-related protein 1 (DRP1) is interconnected with and induced by HIF-1a [8,23,57], the inhibition of mitochondrial fission might offer a comparable target to reduce inflammation and restore bone mineralization. Because of mitochondrial fission’s central role in several cellular pathways, it offers an optimal lever to control the physiology of the cell, both for pathogens and medical agents. In the hypoxic setting, it has been demonstrated that the experimental inhibition of mitochondrial fission has cell-saving effects. It has been shown that experimental inhibition of DRP1 with mitochondrial-division-inhibitor 1 (Mdivi-1) leads to improved cell survival and even reduced infarct size in ischaemic hearts in mice [27,58,59]. It is important to determine the potential value of mitochondrial fission-inhibition in the treatment of chronic osteomyelitis as well. In the course of the treatment of chronic osteomyelitis, bone defect reconstruction using biomaterials as bone substitutes is often necessary. These biomaterials are regularly combined with antibiotics [2]. In a similar way, fission inhibitors could potentially also be added to the substitutes. This should be evaluated via in vitro and animal models.

### 3.3. The Role of Intracellular Staph. aureus in Chronic Osteomyelitis

Lastly, our aim was to quantify the presence of bacteria internalized into bone cells. Neither the light microscopic slides nor the electron microscopic images rendered evidence of intracellular bacteria. Usually bacteria can be detected using light microscopy alone [60]. To rule out the prevalence of intracellular SCVs consisting of *Staph. aureus*’ smaller and more resistant phenotype, we utilized transmission electron microscopy. Compelling data exist regarding *Staph. aureus*’ potential to invade cells, including osteoblasts [61], and evade the immune system [6,62]. The clinical evidence, however, is rather scarce, as only one sole case report exists [63]. Since bone samples are routinely histologically examined during osteomyelitis diagnostics [64], there ought to be more evidence of internalized bacteria if they indeed play a decisive role. Although further investigation is needed, our results also challenge the clinical relevance of intracellular *S. aureus* in chronic osteomyelitis.

### 3.4. Limitations

Some limitations to this study are the limited total number of patients, and the small sample size and inhomogeneity of both groups (Table 1). Furthermore, the results depend on the expertise of the performing surgeon and the exact localization of the acquired tissue. Additionally, the quality of sample preparation also has an impact on the quality of the results.

## 4. Materials and Methods

This study was conducted in accordance with the declaration of Helsinki and was approved by the local ethical review board (reference number: 20_1680_3-101). Sample collection took place from 1 January 2021 to 30 March 2022. The bone samples were acquired during surgical debridements with the patients’ informed consent. The samples were consecutively taken from patients aged 14 or older with chronic osteomyelitis [2], chronic prosthetic joint infection (PJI) of the knee or hip according to the criteria of the European Bone and Joint Infection Society (EBJIS) [65] or a chronic fracture-related infection (FRI) [66] of the lower limb [67]. For inclusion, patients had to be diagnosed with *S. aureus* as an infection causing agent at least once throughout their medical history. Following the pathohistological confirmation of chronic osteomyelitis, patients were included in the study. Control samples were consecutively harvested from non-infected patients planned for iliac crest bone transplantation after their informed consent. Patients were excluded from the control group in the case of systemic conditions that could impede overall mitochondrial function such as inflammation, diabetes or degenerative disorders.

The ultrastructural inclusion criteria for osteocytes were the location within bone lacunae and the absence of cytosolic granolucytic granules [68]. The ultrastructural inclusion criteria for osteoblasts were the proximity to bone, the prevalence of typical abundant dilated rough endoplasmic reticulum, an extensive Golgi apparatus and the absence of granulocytic granules [69]. The inclusion criterion for mitochondria was the presence of a continuous double membrane with invaginations of the inner mitochondrial membrane forming so-called cristae [70]. 

The exclusion criteria for the infectious group were the lack of bacteria following microbiological diagnostics as well as the absence of histopathological correlates of osteomyelitis [71]. The exclusion criteria for the non-infectious control group were the detection of bacteria during microbiological diagnostics or the presence of histopathological correlates of degradation, inflammation or necrosis [71]. 

For electron microscopic analysis of mitochondria from osteoblasts and osteocytes bone samples were primarily fixated in formalin. After primary fixation the samples were transferred to a buffered aqueous glutaraldehyde solution. Alternatively, Karnovsky fixative can be used, which contains buffered paraformaldehyde as well as glutaraldehyde. Following sufficient fixation, the samples are embedded using the LYNX microscopy tissue processor (Reichert-Jung, Wetzlar, Germany). This process involves post-fixation with osmium tetroxide, dehydration and infiltration with EPON, respectively. Semi-thin sections (0.75 µm) were cut using the Reichert Ultracut S Microtome (Leica-Reichert, Wetzlar, Germany) and were stained with toluidine blue and basic fuchsine for the selection of relevant areas via light microscopy. Ultra-thin sections (80 nm), which are cut using the same microtome, are placed on copper grids. These grids were priorly coated with carbon-steamed formvar. The samples were then contrasted with aqueous 2% uranyl-acetate and 2% lead-citrate solution for 10 min each. Electron-microscopy was then performed using the LEO 912AB electron-microscope (Zeiss, Oberkochen, Germany), equipped with a side-mounted 2kx2k CCD-camera (TRS Tröndle, Moorenweis, Germany). Following image acquisition, mitochondrial size, area and circumference were measured using the iTEM software (Olympus Soft Imaging Solutions, Münster, Germany). The data in this study were analyzed with unpaired two-tailed Welch’s unequal variances t-tests for two groups. The data are expressed with means ± s.d. Differences were considered significant at *p* < 0.05. The statistical analysis was performed using the statistics software GraphPad Prism 9.0 for Windows (GraphPad Software, San Diego, CA, USA).

## 5. Conclusions

In conclusion, our results suggest that mitochondrial function and dynamics in human osteoblasts and osteocytes are altered during chronic osteomyelitis in the sense of increased mitochondrial fission. The results indicate an important role of mitochondrial dysfunction in the pathogenesis of chronic osteomyelitis and reveal an alternative treatment approach for this not uncommon and difficult-to-treat disease. Since mitochondrial fragmentation and dysfunction are closely related to the exacerbation of ROS and cell death or apoptosis, they offer an enticing target to improve bone cell survival in chronic osteomyelitis. Recent research efforts elucidated promising new perspectives on therapeutic approaches targeting mitochondria. In this context, it has been demonstrated that the inhibition of mitochondrial fission and transplantation of autologous mitochondria can have cell-saving effects. It is vital to investigate whether these strategies are transferrable to chronic osteomyelitis and bone metabolism. Fission inhibitors could be utilized as adjuncts to bone substitutes to treat or even prevent chronic osteomyelitis.

## Figures and Tables

**Table 1:** List of included patients.

Patient	Osteoblasts Analyzed	Osteocytes Analyzed	Age	Gender	Entity	Location	CCI *	Most Recent Pathogen
**1**	14	6	62	Male	OM	Tibia	4	*MSSA*
**2**	1	6	50	Female	OM	First metatarsal	2	*MSSA; Serratia ureilytica*
**3**	4	8	83	Male	PJI	Femur	2	*MSSA*
**4**	3	16	63	Male	FRI	Femur	1	*Staph. haemolyticus; Staph. epidermidis; VRE*
**5**	8	7	72	Male	PJI	Femur	6	*Staph. epidermidis*
**6**	1	14	56	Male	FRI	Calcaneus	0	*MSSA; Bacillus cereus*
**7**	4	3	59	Male	PJI	Tibia	1	*MSSA; Morganella morganii; E. coli*
**8**	1	3	66	Female	FRI	Tibia	0	*Mycobacterium abscessus*
**9**	7	11	57	Male	FRI	Tibia	2	*Staph. epidermidis*
**10**	14	3	17	Male	OM	Femur	0	*MSSA*
**11**	8	3	40	Male	FRI	Femur	0	*MSSA; Stenotrophomonas maltophilia*
**12**	4	4	71	Male	PJI	Femur	1	*Staph. epidermidis; Entero-coccus faecium*
**13**	0	9	14	Male	OM	Tibia	1	*MRSA*
**14**	3	0	71	Male	PJI	Femur	5	*Streptococcus agalactiae*
**Control**								
**1**	7	15	68	Female	-	Femur	1	*-*
**2**	5	2	53	Male	-	Tibia	0	*-*
**3**	2	11	67	Male	-	Pelvis	0	*-*
**4**	2	6	41	Male	-	Femur	0	*-*

* Charlson-Comorbidity-Index (CCI) as per [28]. *MSSA* = Methicillin-susceptible *Staphylococcus aureus*. *MRSA* = Methicillin-resistant *Staphylococcus aureus*. *VRE* = Vancomycin-resistant *Enterococcus*. *Staph*. = *Staphylococcus*. *E*. = *Escherichia*.

## Data Availability

The data that support the findings of this study are available from the corresponding author, C.B., upon reasonable request.
